# Feasibility and effectiveness of electrochemical dermal conductance measurement for the screening of diabetic neuropathy in primary care. DECODING Study (*Dermal Electrochemical Conductance in Diabetic Neuropathy*). Rationale and design

**DOI:** 10.1097/MD.0000000000010750

**Published:** 2018-05-18

**Authors:** Juan J. Cabré, Teresa Mur, Bernardo Costa, Francisco Barrio, Charo López-Moya, Ramon Sagarra, Montserrat García-Barco, Jesús Vizcaíno, Immaculada Bonaventura, Nicolau Ortiz, Gemma Flores-Mateo

**Affiliations:** a Jordi Gol Primary Care Research Institute, Catalan Health Institute, Primary Health Care Division, Reus-Barcelona, Catalonia; b Mútua Terrassa Primary Care, Terrassa, Barcelona; c Department of Neurology, Hospital Universitari Mútua Terrassa, Terrassa, Barcelona; d Department of Neurology, Hospital Universitari Sant Joan de Reus, Reus, Tarragona, Spain.

**Keywords:** dermal electrochemical conductance, diabetes mellitus, Neuropathy, Primary Care, screening, sudomotor reflex

## Abstract

Diabetes mellitus is the leading cause of polyneuropathy in the Western world. Diabetic neuropathy is a frequent complication of diabetes and may have great clinical transcendence due to pain and possible ulceration of the lower extremities. It is also a relevant cause of morbidity and mortality in patients with diabetes. Although the cause of polyneuropathy in patients with diabetes is only partially known, it has been associated with chronic hyperglycemia suggesting the possible etiopathogenic implication of advanced glycosylation end products. The strategy of choice in the medical management of diabetic neuropathy is early detection since glycaemic control and the use of certain drugs may prevent or slow the development of this disease. Diabetic neuropathy most often presents with a dysfunction of unmyelinated C-fibers, manifested as an alteration of the sweat reflex of the eccrine glands. This dysfunction can now be demonstrated using a newly developed technology which measures dermal electrochemical conductivity. This noninvasive test is easy and cost-effective. The aim of the present study is to evaluate the feasibility and effectiveness of dermal electrochemical conductance measurement (quantitative expression of the sudomotor reflex) as a screening test for the diagnosis of diabetic neuropathy in patients in primary care.

## 1 Introduction

The prevalence of diabetes mellitus (DM) is very high in Spain, being approximately 14% according to oral glucose tolerance test (OGTT) results.^[1]^ The management of DM requires a significant consumption of healthcare resources, mainly in relation to the care of vascular complications. Among the late microvascular events which may develop in patients with DM, polyneuropathy (PN) is the most common and disabling, and is the leading cause of morbidity and mortality in these patients.^[2]^ Indeed, in Spain, the leading cause of neuropathy is DM, with its prevalence increasing with the presence of DM and other risk factors such as obesity.^[3]^ PN is defined as the presence of symptoms and/or signs of peripheral nerve dysfunction in people with DM after the exclusion of other possible causes.^[4]^ The Toronto Panel Consensus on PN defined this disorder as “symmetrical, depending on large fibers, sensory-motor attributable to metabolic and microvessel disorders, as a result of chronic hyperglycemia and other risk factors.”^[2]^ In patients with PN, thin fibers (autonomic system—sweating) and thermal and tactile sensitivity are first affected, followed by the involvement of large fibers, presenting an altered vibrating sensation which eventually alters electromyography (EMG) patterns. Therefore, dysfunction of sweat reflex in small distal fibers is one of the earliest changes to be detected in these patients.^[5]^ The most common clinical presentation of PN is distal symmetric polyneuropathy (DSPN), being predominantly sensory in 80% of cases.^[3]^ Pain is the most important symptom, being described as burning or flashing, lancinanting, deep, and with frequent exacerbations during rest.^[4]^ Pain often affects the quality of life of these patients, and it is a frequent cause of depression and/or anxiety.^[6]^ Moreover, some patients may develop hypoesthesia, which may lead to severe foot lesions.^[7]^

The prevalence of DSPN varies greatly according to the population, definition, and detection method. The Rochester study, including >64,000 patients, reported the prevalence of PN to be between 66% and 59% for type1 DM and type 2 DM, respectively.^[8]^ The 3rd report of the Technical Study Group of Diabetes of the World Health Organization (WHO) described a prevalence of 40%,^[8]^ and 50% in patients with >25 years of DM evolution. Pirart et al^[9]^ reported a prevalence ranging from 25% to 48%,^[7,10–17]^ whereas in Spain, Cabezas-Cerrato et al published a figure of 24.1%.^[11]^ DSPN-related factors are age, DM duration, metabolic control, male gender, acute myocardial infarction, hyperlipidaemia (especially hypertriglyceridaemia), smoking, and general cardiovascular risk factors.^[2,15,16,18]^ Puig et al^[15]^ also included urinary albumin excretion as a risk factor of presenting DSPN. The diagnosis of DSPN is commonly made based on signs and symptoms and usually includes the use of several questionnaires such as the Neuropathy Disability Score (NDS), the Neuropathy Symptoms Score (NSS) and the Michigan Neuropathy Instrument (MNI). These questionnaires are easy to perform and are reproducible, sensitive, and adequate for use in a screening program.^[16]^ In addition, we included a short scale (UENS—Utah Early Neuropathy Scale) to screen early neuropathy.^[17]^ This sensitive, fast and practical test, has 5 items and their score ranges from 0 to 42 points.

There are many confirmatory tests, including measurements of nerve conduction velocity (EMG) and biothesiometry or skin biopsy. However, those most commonly used are the measurement of altered sensations using a vibrating tuning fork with 128 Hz and/or pressure with Semmes–Weinstein 5:07 monofilament.^[18]^ Monofilament testing (MFT) is widely accepted and recommended by all scientific societies because of its validity, predictive risk, efficiency, and simplicity. Feng et al^[19]^ reported that MFT has a sensitivity of 57% to 93%, a specificity of 75% to 100%, a positive predictive value of 36% to 94%, and a negative predictive value of 84% to 100% compared with the measurement of nerve velocity by EMG. Although electrophysiological measures are more objective and reproducible, they are limited in that they only detect dysfunction based on the presence of thicker and faster (myelinated) fibers and show their involvement later. Consequently, EMG is a specific, albeit very insensitive, test.

Recently developed noninvasive techniques are more reproducible and reliable for the detection of early dysfunction of small fibers. One of these new techniques involves the measurement of *dermal electrochemical conductance* (DEC) or sudomotor dysfunction index and has been evaluated by well-designed studies which support its use as a screening test.^[20]^

Ramachandran et al ^[21]^ studied the use of DEC to detect diabetes and other disorders of glucose metabolism. In a study on the use of DEC, Casellini et al ^[5]^ applied a PN test which showed a low sensitivity of 78% and a specificity of 92% in diabetic patients without neuropathy compared to other subjects with neuropathy and a control group. In this latter study, correlation with clinical parameters showed adequate reproducibility of the results, particularly in regards to the measurements of the feet.^[5]^ Several other studies^[22]^ also obtained significantly lower DEC values on comparing diabetic patients and controls. In a study of patients following a 12-month program of intense physical activity, Raisanen et al ^[23]^ observed a greater improvement in DEC compared to weight, waist circumference, or maximum oxygen volume (VO_2_ max). Therefore, taking into account the large number of methods used and the learning curve required to correctly implement these techniques as well as the absence of consensus as to which method is the most adequate to diagnose DSPN, the aim of this study is to validate the usefulness of DEC measurement in the early diagnosis of DSPN compared with traditional techniques in the primary care setting.

## 2 Hypothesis and objectives

The hypothesis of our study is that the measurement of DEC is feasible, sensitive, and specific and more or equally effective to other techniques commonly used in the initial screening of diabetic neuropathy in primary care.

### 2.1 Main objective

To evaluate the feasibility, effectiveness and performance of a new technique which measures DEC (sudomotor reflex) in the screening of diabetic neuropathy in primary care.

### 2.2 Specific objectives

1. Determine the performance of DEC (quantitative assessment of sudomotor reflex) as a tool for the screening of diabetic neuropathy in primary care compared with the Semmes–Weinstein 5:07 MFT when an EMG is used to confirm the presence of diabetic peripheral neuropathy in patients with prediabetes and type 2 diabetes.
2. Determine the performance of DEC (quantitative assessment of sudomotor reflex) as a tool for screening diabetic neuropathy in Primary Care compared with the Semmes–Weinstein 5:07 MFT when the UENS (Utah Early Neuropathy Score) is used to confirm the presence of diabetic peripheral neuropathy in patients with prediabetes and type 2 diabetes.
3. Estimate the cost and cost-effectiveness of the use of DEC in the screening of early diabetic neuropathy in primary care.

## 3 Methods

### 3.1 Design

We will perform a blind, prospective study comparing DEC (sudomotor reflex) (Sudoscan, Impeto Medical, France), EMG, the Semmes–Weinstein 5:07 MFT (10 g), the sensitivity of a vibrating tuning fork 128 Hz, the NDS score, and the UENS score in a consecutive series of patients treated in primary care.

### 3.2 Sites

The primary care teams of Terrassa-Sud and other partners belonging to the Mutua Terrassa reference hospital and those of the University Hospital Sant Joan de Reus reference hospital.

### 3.3 Study subjects

We will consecutively include patients with type 2 DM over 40 years of age, with or without symptoms of neuropathy, attended in primary care. We will also include the following 2 groups of patients matched by age and sex: one including patients with prediabetes (intermediate alterations of glucose metabolism defined as impaired fasting glucose [IFG]) and/or impaired glucose tolerance (IGT) determined by OGTT after 2-h 75 g oral glucose administration and another including patients without glucose alterations (normal glucose tolerance) (control group).

Three main diagnostic categories (normal, prediabetes, and diabetes) were defined using the WHO criteria based on 2-h postload glucose [<7.8 (140 mg/dL), 7.8–11.0 mmol/L (140–200 mg/dL)] and/or fasting plasma glucose (6.1–6.9 mmol/L; 110–126 mg/dL) and >11.1 mmol/L (>200 mg/dL), respectively.

The exclusion criteria are type 1 DM, upper or lower limb amputation (except phalanges), diagnosis of neuropathy not related to diabetes, neuropathy by entrapment, use of psychoactive substances, chronic alcoholism, malnutrition; treatment with beta-blockers, presence of terminal disease, or life expectancy <3 years. Pregnancy will be ruled out in women (negative pregnancy test) and a possible history of gestational diabetes will also be taken into account.

The study period is from January 1, 2017 to November 30, 2019.

### 3.4 Sample size

To estimate the validity and performance of a screening test that showed a sensitivity of 82%, a precision of 9%, and a confidence interval of 95% (95% CI), considering a loss percentage of 20%, this study will include a total of 160 participants. The proportion of diabetes/prediabetes/normal glucose tolerance will be 2:1:1. The contribution by centers will be 66% from the Mutua de Terrassa (106 cases) and 34% from Reus (54 cases).

### 3.5 Variables and dynamic data collection

After verifying the inclusion criteria and receiving written informed consent to participate, during the *first visit* to the primary care centers the medical history of the patient will be obtained and a physical examination will be performed using the MFT and the NDS and UENS questionnaires will be given to screen for PN. The patient will also undergo DEC quantification using the Sudoscan device.

The following variables were collected:

- Age, sex, and country of origin.
- Family history of type1 or type2 DM: (yes/no).
- In diabetic population, length of diabetes evolution (in years) since diagnosis.
- Toxic habits: smoking (active smoker/ex smoker/nonsmoker); drinking (teetotal, occasional drinker, habitual drinker).
- Other complications of diabetes and year of diagnosis (retinopathy, ischemic heart disease, peripheral vascular disease, cerebral vascular disease, and diabetic nephropathy) will be collected from the patient medical records.
- Diabetes treatment.
- Other pharmacological treatments related to hypotension, hypolipidemia, arterial thrombosis, among others.

On physical examination the following variables will be collected:

- Anthropometric data: Height (cm), weight (kg) (using validated clinical scale with stadiometer in light clothing), waist circumference (cm) (using a flexible measuring tape), measuring the point between the navel and upper iliac crest.
- Systolic/diastolic blood pressure; presence of pulses in pedia/posterior tibial arteries. Blood pressure (mm Hg) measurements will be made 3 times while seated after 30 minutes of rest using a validated automatic device, with each measure being made ≥1 minute apart. The data recorded will be the mean of the second and third readings.

Other data to be collected include:

- Blood/urine tests: OGTT (except in known DM); glycosylated hemoglobin A1c (high-resolution liquid chromatography [HPLC]), glomerular filtration rate (modification of diet in renal disease [MDRD]/Chronic Kidney Disease Epidemiology Collaboration [CKD-EPI]); lipid profile (total cholesterol, triglycerides and LDL and HDL fractions using the cholesterol oxidase-phenol + aminophenazone (CHOD-PAP) method. These latter parameters should have been determined no more than 6 months previously. If not the determinations should be made in the reference laboratory.
- A score of 8 out of 8 with the Semmes–Weinstein 5:07 MFT (10 g) will be considered as sensitive.
- A NDS score ≥6 points will be considered as the presence of PN.
- A UENS score ≥10 points will be considered as the presence of PN.
- The determination of vibration sensitivity will be performed using a 128 Hz Rydel–Seiffer tuning fork. The test will be considered positive for PN when vibration of the tuning fork is not perceived when applied to the thumb or external maleolus.

In the *second visit*, done at the reference hospital, a neurologist blinded to previous test results, will perform neurographyc test, including sensory conduction study of the median, ulnar and sural nerves, and motor conduction study of the deep peroneal nerve. The studied variables will be amplitude of compound muscle action potential and distal latency of the motor nerves, and amplitude and distal latency of sensory nerves. DEC determination and the other neuropathy screening and electrophysiological tests will take no longer than 1 month.

The *third visit* will be carried out in the primary care center where the results of the previous visit will be recorded and the patient will be informed of the results and the diagnosis.

The cost-effectiveness of the different diagnostic methods studied will be evaluated and the incremental cost method to detect new cases compared with traditional methods will be calculated. In addition, a computer-simulated model will determine the long term as well as the direct and indirect costs of the different methods.

### 3.6 Statistical analysis

The χ^2^ test will be used to analyze qualitative variables and the Student *t* test will be performed for quantitative variables. Logistic regression will be used to identify predictors of diabetic neuropathy. Dependent variables (response) will include the presence of diabetic neuropathy diagnosed by EMG or the NDS/UENS questionnaires. We will compare the performance of DEC with that of the MFT as screening tests of PN. To determine the validity and reliability of DEC, we will calculate the sensitivity, specificity, the positive and negative predictive values, and the positive and negative likelihood ratios. A ROC curve will be used and the area under the curve will be calculated. A *P* <.05 will be considered as statistically significant. The analysis will be performed with the statistical packages STATA/SE 12.0 and R for Windows.

### 3.7 Limitations of the study

The main limitation of the present study is accurate diagnosis of diabetic neuropathy because some studies have shown that some cases of diabetic neuropathy present no alterations in the EMG. Indeed, several considerations should be taken into account. First, the EMG test is more specific, albeit not very sensitive, showing positive results in advanced stages of PN. The fingerboard and the NDS questionnaire are commonly used for the diagnosis of diabetic neuropathy, probably because the NDS is carried out before EMG and is actually often used to avoid the need for EMG. Therefore, both the EMG and the NDS score, which mainly assess the dysfunction of myelinated fibers, provide a good profile for diagnostic confirmation. On the contrary, both the DEC and the MFT are able to diagnose and stage diabetic neuropathy earlier than the previous 2 tests by the detection of unmyelinated fiber dysfunction. But another limitation is that MFT is a good test for prediction of a foot ulcer but is certainly insensitive for the detection of early neuropathy. For this reason, we think about the UENS because of its sensitivity to early sensory loss and ability to record modest anatomic change in sensory function, considering patients with milder neuropathy. Therefore, for purposes of simplification and taking into account the possible limitations, we will compare the effectiveness of the measurement of DEC and the use of MFT as diagnostic tools in primary care according to whether the true diagnosis is achieved by the EMG or the score of the NDS or UENS questionnaires.

Obviously, there are another tests to detect early neuropathy, that is, the Norfolk QOL-DN scale, but this questionnaire is not practical in the clinical scenario. Certainly, this test is a good tool to detect unaware neuropathy in patients with diabetes.^[24]^ This study used the Norfolk QOL-DN scale on 25,000 patients with diabetes and found 6600 patients who were not aware of their neuropathy nor did their physicians know.

Another limitation of this study is the possibility of introducing a selection bias, and thus, we will consecutively include patients with type 2 DM, excluding patients with type 1 DM. We will also include a group of patients without DM or other glucose homeostasis disorders matched by age and sex to a 2:1:1 ratio (80 with DM, 40 with prediabetes and 40 with normal glucose tolerance).

## 4 Quality aspects

This project will promote a strategy to determine whether the implementation of a new technique simplifies the detection of a major complication of diabetes, such as neuropathy. Primary care is clearly the most appropriate setting for the screening of this complication. Indeed, the earlier the diagnosis the better the prognosis. This strategic action on health is directly linked to:

1. New medical technologies to promote personalized medicine based on the profiles of the individuals and not the disease.
2. Translational and clinical research, evidence-based scientific and technological knowledge.
3. Validation of a promising new technology for early detection of diabetic neuropathy in primary care.
4. Application of evidence in clinical practice. This is a validation study of diagnostic technology implemented in real life conditions which would provide additional knowledge for everyday clinical practice. It is of note that this study will also determine the cost-effectiveness of this new tool which is essential for the validation of new technologies aimed at different healthcare areas such as primary care.

In addition, this project will establish synergies at different levels such as primary care and neurology, and the results can be shared with a number of centers, primary care areas, and different scientific societies involved in the comprehensive care of patients with diabetes. The results of previous studies support the feasibility of DEC in primary care, although further studies should be aimed at complementing and extending our hypothesis to different types of candidates and determining the cost-effectiveness of the systematic use of DEC.

## 5 Technical and collaborative aspects

The number of centers involved is limited, including primary care centers of the Catalan Institute of Health (ICS) of Reus (Tarragona) and the Mútua Terrassa (Barcelona). In addition, IDIAP Jordi Gol and the Pere Virgili Health Research Institute—Hospital de Sant Joan (Reus), and IDIAP Jordi Gol and Mútua Terrassa have signed a cooperation agreement regarding research in the field of primary care.

## Author contributions

All authors substantially contributed to designing the study protocol or to data analysis and interpretation, and to drafting or revising the article. Specifically, JJC, TM, and BC formulated the research question, designed the study (by cooperating with FB, RS, JV, and GFO), and wrote the research proposal as well as the first draft of the manuscript. JJC, TM, and BC discussed the proposal and his approach to the reality of primary care. BC, TM, FB, CLM, MGB, IB, NO, and GFO critically revising the proposal for scientifical content.

All authors contributed to the revision of the manuscript, read and approved the final version.

**Conceptualization:** Juan José Cabré Vila, Teresa Mur, Bernardo Costa Pinel, Francisco Barrio, Ramon Sagarra, Jesus Vizcaino, Immaculada Bonaventura, Nicolau Ortiz, Gemma Flores-Mateo.

**Data curation:** Juan José Cabré Vila, Charo Lopez-Moya, Ramon Sagarra, Montserrat García-Barco, Immaculada Bonaventura, Nicolau Ortiz.

**Formal analysis:** Bernardo Costa Pinel, Immaculada Bonaventura.

**Funding acquisition:** Bernardo Costa Pinel.

**Investigation:** Juan José Cabré Vila, Teresa Mur, Bernardo Costa Pinel, Francisco Barrio, Montserrat García-Barco, Jesus Vizcaino, Gemma Flores-Mateo.

**Methodology:** Juan José Cabré Vila, Teresa Mur, Bernardo Costa Pinel, Francisco Barrio, Jesus Vizcaino, Immaculada Bonaventura, Nicolau Ortiz, Gemma Flores-Mateo.

**Project administration:** Bernardo Costa Pinel.

**Resources:** Teresa Mur, Bernardo Costa Pinel, Francisco Barrio.

**Supervision:** Juan José Cabré Vila, Bernardo Costa Pinel, Francisco Barrio, Ramon Sagarra, Montserrat García-Barco, Immaculada Bonaventura, Gemma Flores-Mateo.

**Validation:** Juan José Cabré Vila, Teresa Mur, Bernardo Costa Pinel, Charo Lopez-Moya, Ramon Sagarra, Immaculada Bonaventura, Nicolau Ortiz, Gemma Flores-Mateo.

**Visualization:** Juan José Cabré Vila, Francisco Barrio, Charo Lopez-Moya, Ramon Sagarra, Montserrat García-Barco, Jesus Vizcaino.

**Writing** – **original draft:** Juan José Cabré Vila, Teresa Mur, Bernardo Costa Pinel, Francisco Barrio, Immaculada Bonaventura, Nicolau Ortiz, Gemma Flores-Mateo.

## References

[1] Soriguer F Goday A Bosch-Comas A Prevalence of diabetes mellitus and impaired glucose regulation in Spain: the Di@betes Study. Diabetologia 2012;55:88–93. 21987347 10.1007/s00125-011-2336-9 PMC3228950

[2] Tesfaye S Selvarajah D Advances in the epidemiology, pathogenesis and management of diabetic peripheral neuropathy. Diabetes Metab Res Rev 2012;28 Suppl. 1:8–14. 10.1002/dmrr.2239 22271716

[3] Said G Diabetic neuropathy—a review. Nat Clin Pract Neurol 2007;3:331–40. 17549059 10.1038/ncpneuro0504

[4] Samper Bernal D Monerris Tabasco MM Homs Riera M Etiología y manejo de la neuropatía diabética dolorosa. Rev Soc Esp Dolor 2010;17:286–96. [article in Spanish].

[5] Casellini CM Parson HK Richardson MS Sudoscan, a noninvasive tool for detecting diabetic small fiber neuropathy and autonomic dysfunction. Diabetes Technol Ther 2013;15:948–53. 23889506 10.1089/dia.2013.0129 PMC3817891

[6] Gore M Brandenburg NA Dukes E Pain severity in diabetic peripheral neuropathy is associated with patient functioning, symptom levels of anxiety and depression, and sleep. J Pain Symptom Manage 2005;30:374–85. 16256902 10.1016/j.jpainsymman.2005.04.009

[7] Tesfaye S Stevens LK Stephenson JM Prevalence of diabetic peripheral neuropathy and its relation to glycaemic control and potential risk factors: the EURODIAB IDDM Complications Study. Diabetologia 1996;39:1377–84. 8933008 10.1007/s001250050586

[8] Vidal MA Martínez-Fernández E Martínez-Vázquez de Castro J Neuropatía diabética. Eficacia de la amitriptilina y de la gabapentina. Rev Soc Esp Dolor 2004;11:490–504. [article in Spanish].

[9] Pirart J Diabetes mellitus and its degenerative complications: a prospective study of 4,400 patients observed between 1947 and 1973. Diabetes Care 1978;1:168–88.

[10] Dyck PJ Kratz KM Karnes JL The prevalence by staged severity of various types of diabetic neuropathy, retinopathy, and nephropathy in a population-based cohort The Rochester Diabetic Neuropathy Study. Neurology 1993;43:817–1817. 8469345 10.1212/wnl.43.4.817

[11] Cabezas-Cerrato J The prevalence of clinical diabetic polyneuropathy in Spain: a study in primary care and hospital clinic groups. Diabetologia 1998;41:1263–9. 9833931 10.1007/s001250051063

[12] Gómez MAGM Estudio de la conduccion nerviosa en pacientes con diabetes mellitus tipo 2. Rev Peru Endocrinol Metab 1998;IV:23–33. [article in Spanish].

[13] Lu B Hu J Wen J Determination of peripheral neuropathy prevalence and associated factors in Chinese subjects with diabetes and pre-diabetes—ShangHai Diabetic neuRopathy Epidemiology and Molecular Genetics Study (SH-DREAMS). PloS One 2013;8:e61053. 23613782 10.1371/journal.pone.0061053 PMC3628856

[14] Ziegler D Papanas N Rathmann W Evaluation of the Neuropad sudomotor function test as a screening tool for polyneuropathy in the elderly population with diabetes and pre-diabetes: the KORA F4 survey. Diabetes Metab Res Rev 2012;28:692–7. 22949335 10.1002/dmrr.2340

[15] Puig ML Aguirre DR Rodríguez MC Neuropatía periférica de los miembros inferiores en diabéticos tipo 2 de diagnóstico reciente. Av Endocrinol 2006;22:149 [article in Spanish].

[16] Calle Pascual AL Runkle Vega I Díaz Pérez JA Técnicas de exploración. Av Diabetol 2006;22:42–9. [article in Spanish].

[17] Singleton JR Bixby B Russell JW The Utah Early Neuropathy Scale: a sensitive clinical scale for early sensory predominant neuropathy. J Peripher Nerv Syst 2008;13:218–27. 18844788 10.1111/j.1529-8027.2008.00180.x

[18] Kanji JN Anglin RE Hunt DL Does this patient with diabetes have large-fiber peripheral neuropathy? JAMA 2010;303:1526–32. 20407062 10.1001/jama.2010.428

[19] Feng Y Schlösser FJ Sumpio BE The Semmes Weinstein monofilament examination as a screening tool for diabetic peripheral neuropathy. J Vasc Surg 2009;50:675–82. 19595541 10.1016/j.jvs.2009.05.017

[20] Gordon Smith A Lessard M Reyna S The diagnostic utility of Sudoscan for distal symmetric peripheral neuropathy. J Diabetes Complications 2014. 10.1016/j.jdiacomp.2014.02.013 PMC4219320 24661818

[21] Ramachandran A Moses A Shetty S A new non-invasive technology to screen for dysglycaemia including diabetes. Diabetes Res Clin Pract 2010;88:302–6. 20188429 10.1016/j.diabres.2010.01.023

[22] Mayaudon H Miloche P-O Bauduceau B A new simple method for assessing sudomotor function: relevance in type 2 diabetes. Diabetes Metab 2010;36:450–4. 20739207 10.1016/j.diabet.2010.05.004

[23] Raisanen A Eklund J Calvet J-H Sudomotor function as a tool for cardiorespiratory fitness level evaluation: comparison with maximal exercise capacity. Int J Environ Res Public Health 2014;11:5839–48. 24886754 10.3390/ijerph110605839 PMC4078551

[24] Veresiu AI Bondor CI Florea B Detection of undisclosed neuropathy and assessment of its impact on quality of life: a survey in 25,000 Romanian patients with diabetes. J Diabetes Complications 2015;29:644–9. 25922309 10.1016/j.jdiacomp.2015.04.001

